# Case report: Infratentorial Embryonal Tumor with Abundant Neuropil and True Rosettes (ETANTR) in an 8-month-old Maine Coon

**DOI:** 10.3389/fvets.2022.961056

**Published:** 2022-08-25

**Authors:** Greta Foiani, Maria Teresa Mandara, Antonio Carminato, Erica Melchiotti, Michela Corrò, Marta Vascellari

**Affiliations:** ^1^Laboratory of Histopathology, Istituto Zooprofilattico Sperimentale delle Venezie, SCS3, Legnaro, Italy; ^2^Department of Veterinary Medicine, University of Perugia, Perugia, Italy; ^3^Clinical Diagnostic Laboratory, Istituto Zooprofilattico Sperimentale delle Venezie, SCT3, Legnaro, Italy

**Keywords:** cat, central nervous system, embryonal tumor, multilayered rosettes, infratentorial, ETANTR

## Abstract

An 8-month-old female Main Coon with a history of recurrent behavioral changes and anorexia was presented with sternal recumbency and depression. Within 5 days, the cat progressively worsened with symptoms of stupor and coma and was euthanized. At post-mortem examination, a solid, grayish infratentorial mass located in the midline rostrally to the cerebellum, was observed. Histologically, highly cellular clusters of small-to-medium undifferentiated cells were intermingled with paucicellular areas with fibrillary eosinophilic (neuropil-like) appearance. Numerous multilayered (ependymoblastic) true rosettes were present. The mitotic activity was frequent (up to 15 mitoses/HPF), involving both undifferentiated cells and rosettes. By immunohistochemistry (IHC), tumor cells were diffusely positive for vimentin, variably for synaptophysin, S-100, and NSE, and focally for NeuN; they were negative for GFAP and CK AE1/AE3. The histological and IHC aspects were consistent with an Embryonal Tumor with Abundant Neuropil and True Rosettes (ETANTR). Embryonal neoplasms of the central nervous system (CNS) are characterized by primitive undifferentiated cells, able to develop toward neuronal, glial, ependymal, and mesenchymal lines. Although extremely rare, juvenile embryonal tumors should be considered in the differentials of CNS disorders in young cats.

## Introduction

Embryonal tumors of the central nervous system (CNS) arise from progenitor cells capable of differentiating into different lineages, including neuronal, glial, ependymal and mesenchymal cell lines (1, 2). Although these aggressive tumors occur mainly in infants and young children, they are mostly reported in juvenile and adult domestic animals (3–7).

The classification and nomenclature of embryonal CNS neoplasms has long been controversial, both in veterinary and human neuropathology, because of their poorly differentiated cytological characteristics (8).

The term primitive neuroectodermal tumors (PNET) was used for decades to refer to human CNS tumors arising supratentorially, in the brainstem or spinal cord, composed of undifferentiated or poorly differentiated neuroepithelial cells (9). The 2016 World Health Organization (WHO) classification of CNS tumors removed PNET and other morphological terms from the diagnostic lexicon (10). In the current human WHO 2021 classification, embryonal CNS neoplasms are classified based on a combination of histopathological and molecular features (11). They are divided in two broad groups: medulloblastomas, and other embryonal CNS tumors. Included in the latter is the Embryonal Tumor with Multilayered Rosettes (ETMR), a recently introduced molecular entity (9, 12). Prior to the reclassification of ETMR as a single entity, three different histological variants were recognized, named Embryonal Tumor with Abundant Neuropil and True Rosettes (ETANTR), Ependymoblastoma (EBL) and Medulloepithelioma (MEPL) (10, 12).

Contrarily to human pathology, WHO classification of embryonal CNS tumors of domestic animals still includes the term PNET, with the following categories: medulloblastoma, defined as PNET arising from the cerebellum; non-cerebellar PNET histologically indistinguishable from medulloblastoma; neuroblastoma with neural differentiation; and ependymoblastoma with ependymal differentiation (1).

Embryonal CNS tumors are rarely reported in the veterinary literature (13). Medulloblastomas have been described in dogs, cattle, pigs, rats, and non-human primates with a single report in cats (7, 8, 14–18). Other CNS embryonal tumors are extremely rare and have been described mainly in dogs, with fewer reports in cattle (6, 19–21).

To the authors' knowledge, embryonal CNS tumors other than medulloblastomas have not been reported in cats. Herein we describe the gross, histopathologic and immunohistochemical features of an infratentorial embryonal tumor (non-cerebellar PNET) consistent with the human variant ETANTR, in a young cat.

## Case description

An 8-month-old female Main Coon was presented to the referring veterinarian practitioner with sternal recumbency and depression. The cat had a history of recurrent behavioral changes and anorexia. Within 5 days of hospitalization, the cat progressively worsened with symptoms of stupor and coma and was therefore euthanized.

**Figure 1:**
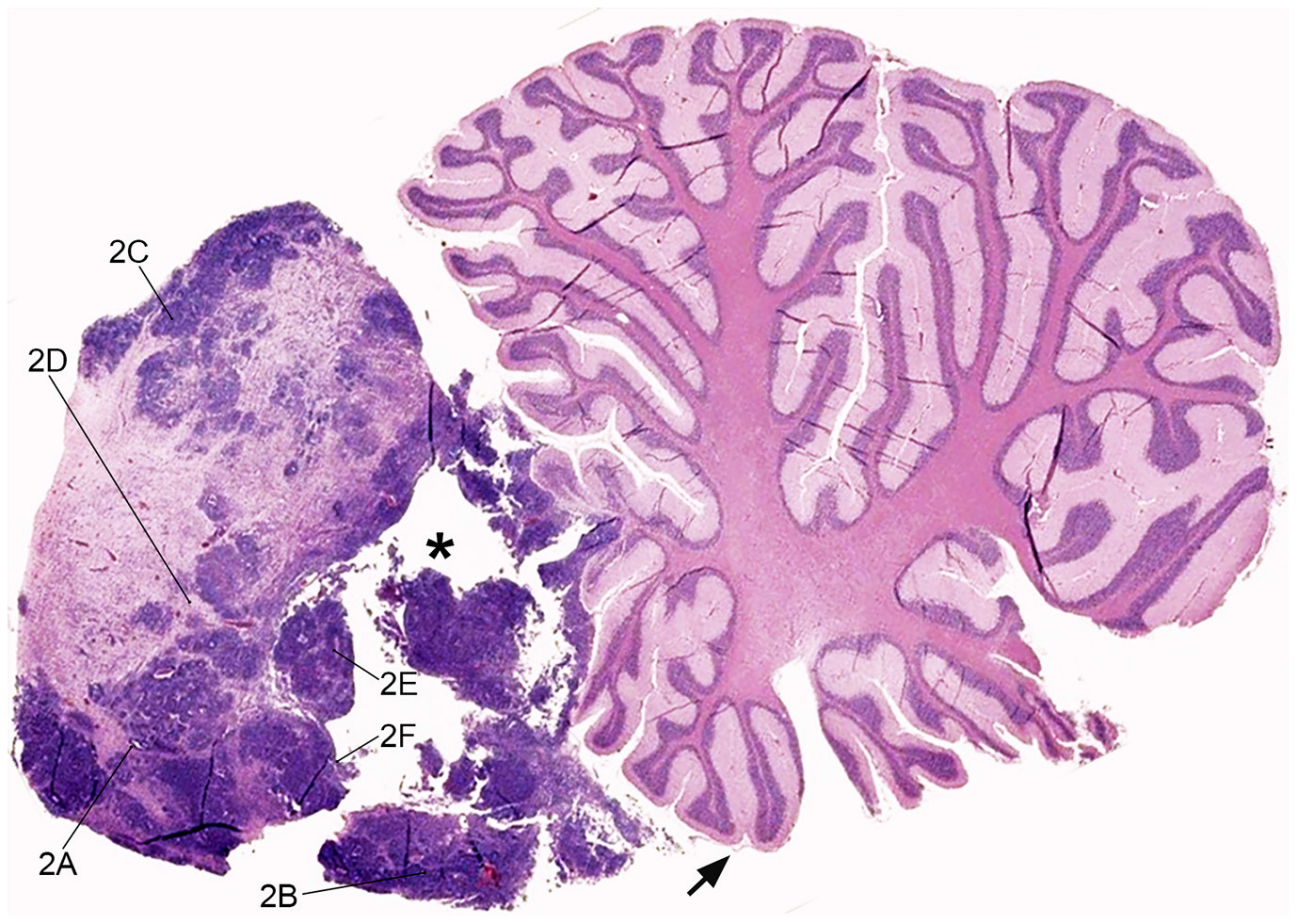
Feline embryonal tumor located rostrally to the cerebellum, sub-gross view. Sagittal para-medial section of the mass, displacing the rostral cerebellar lobe and occupying the rostral fourth ventricle. The neoplasm infiltrates the rostral medullary velum (arrow), the cerebellar leptomeninges, and focally the cerebellar folia. Densely cellular tumor foci are intermingled with paucicellular eosinophilic areas and focally extensive cystic degeneration (asterisk). The lines indicate the region of acquisition of Figure 2 images. Hematoxylin and eosin (H&E) stain.

After necropsy, the brain was fixed in 10% neutral-buffered formalin for 7 days. For neuropathological examination, the brain was firstly sectioned on the longitudinal midsagittal plane; the cerebral hemispheres and the diencephalon were then trimmed in coronal slices, and the cerebellum and the caudal brainstem in longitudinal slices. A solid, grayish infratentorial mass was observed in the midline rostrally to the cerebellum, occupying the rostral part of the fourth ventricle. The mass measured 1.5 cm in width, 1.2 cm in length, and 1.5 cm height. It was macroscopically well-demarcated although it compressed and infiltrated the rostral cerebellar lobe, the rostral medullary velum, the floor of the fourth ventricle, and focally the caudal colliculus (Figure 1). The brain had a normal overall morphology and size. However, the third and lateral ventricles were moderately dilated. Severe lung and hepatic congestion, pulmonary edema, and a single cyst in the left ovary were also observed.

**Figure 2:**
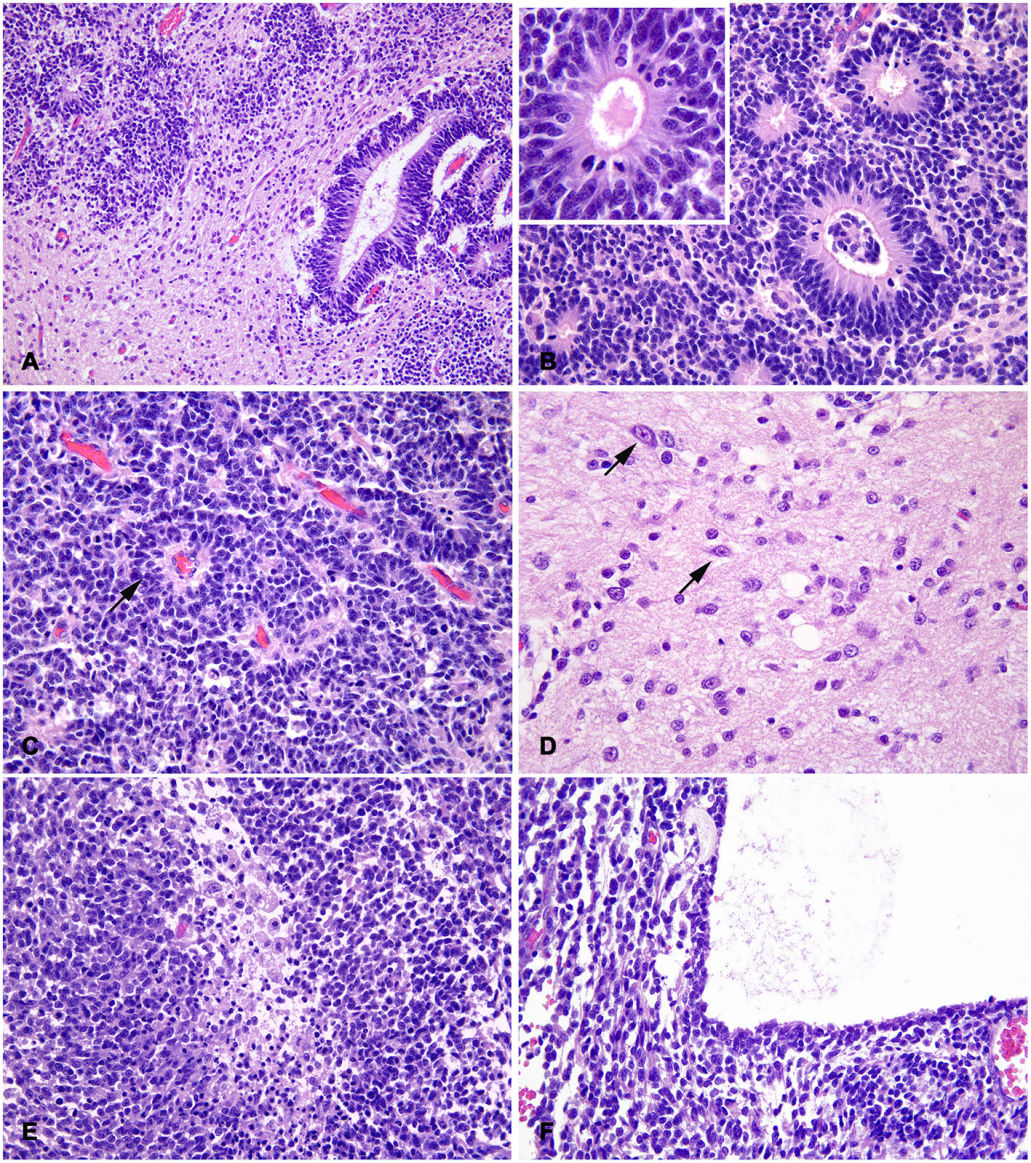
Feline embryonal tumor with multilayered rosettes, neuropil-like areas and foci of neurocytic differentiation; histological aspects. **(A)** The tumor is composed by dense clusters of small-to-medium undifferentiated cells, paucicellular regions with fibrillary eosinophilic (neuropil-like) appearance and multilayered rosettes with slit-like or round lumens. **(B)** Rosettes have multiple peripheral nuclear layers and a central nuclei-free zone with frequent mitotic figures; central lumens are bounded by distinct cell membranes with eosinophilic contouring and debris (insert). **(C)** Irregular chords of undifferentiated cells have scant indistinct cytoplasm and form perivascular pseudorosettes (arrow). **(D)** In neuropil-like areas, rare scattered cells exhibit neurocytic differentiation (arrows). **(E)** Focal necrosis with macrophage infiltration in a densely cellular and highly proliferating area. **(F)** Part of the mass limiting the area of cystic degeneration, characterized by optically empty lumen with sparse eosinophilic debris. H&E stain.

At sub-gross examination, the neoplasm was composed of areas with different cellularity with focally extensive cystic degeneration (Figure 1). Microscopically, dense irregular clusters, bundles and cords of small-to-medium undifferentiated cells with scant cytoplasm and indistinct cell borders were intermingled with paucicellular regions with fibrillary eosinophilic (neuropil-like) appearance (Figure 2A). Numerous multilayered true rosettes were found in both the highly cellular and neuropil-like areas (Figure 2B). Rosettes were characterized by central round or slit-like lumens bounded by distinct cell membranes. Lumens were empty but frequently contained fine irregular granules and/or exhibited delicate eosinophilic contouring of the membrane surface (Figure 2B insert). Nuclei of rosette-forming cells were aligned away from the lumen (ependymoblastic rosettes). Small undifferentiated cells had round/polygonal to elongated nuclei with dense chromatin. Frequently, neoplastic cells also formed palisades around capillaries (perivascular pseudorosettes; Figure 2C). Multilayered rosettes and undifferentiated cells were characterized by brisk mitotic activity (up to 15 mitoses/HPF) and apoptotic bodies. Within rosettes, mitotic figures were mostly seen near the luminal border. In neuropil-like areas, foci of desmoplastic stromal response and rare neurocytic cells were present (Figure 2D). Few small necrotic foci were scattered through the neoplasia (Figure 2E), mostly near cystic degeneration area, containing sparse necrotic debris (Figure 2F).

To better characterize the neoplastic population, 4 μm serial sections of formalin-fixed, paraffin-embedded tissue were submitted to automated (Discovery ULTRA system, Roche, Ventana Medical Systems Inc., Tucson, AZ, USA) immunohistochemistry (IHC) with the primary antibodies anti- cytokeratin (CK) (1:50), vimentin (1:100), synaptophysin (1:10), glial fibrillary acidic protein (GFAP; 1:200), S-100 protein (1:500), Ki67 (1:50), neuron specific enolase (NSE; 1:150), and neuronal nuclear antigen (NeuN; 1:1000). The antibody clone, manufacturers and catalog numbers are specified in Table 1.

**Table 1:** List of primary antibodies tested on the feline embryonal tumor with multilayered rosettes, with manufacturers and catalog numbers.

**Primary antibody, clone**	**Manufacturer**	**Catalog number**
Cytokeratin (CK), AE1/AE3	Dako, Agilent Technologies, Glostrup, Denmark	M3515
Vimentin, V9	Dako, Agilent Technologies, Glostrup, Denmark	M0725
Synaptophysin, DAK-SYNAP	Dako, Agilent Technologies, Glostrup, Denmark	M7315
Glial fibrillary acidic protein (GFAP), 6F2	Dako, Agilent Technologies, Glostrup, Denmark	M0761
S-100 protein (polyclonal)	Dako, Agilent Technologies, Glostrup, Denmark	Z0311
Ki67, MIB-1	Dako, Agilent Technologies, Glostrup, Denmark	M7240
Neuron specific enolase (NSE), BBS/NC/VI-H14	Dako, Agilent Technologies, Glostrup, Denmark	M0873
Neuronal nuclear antigen (NeuN), A60	Millipore, Burlington, MA, USA	MAB377

**Figure 3:**
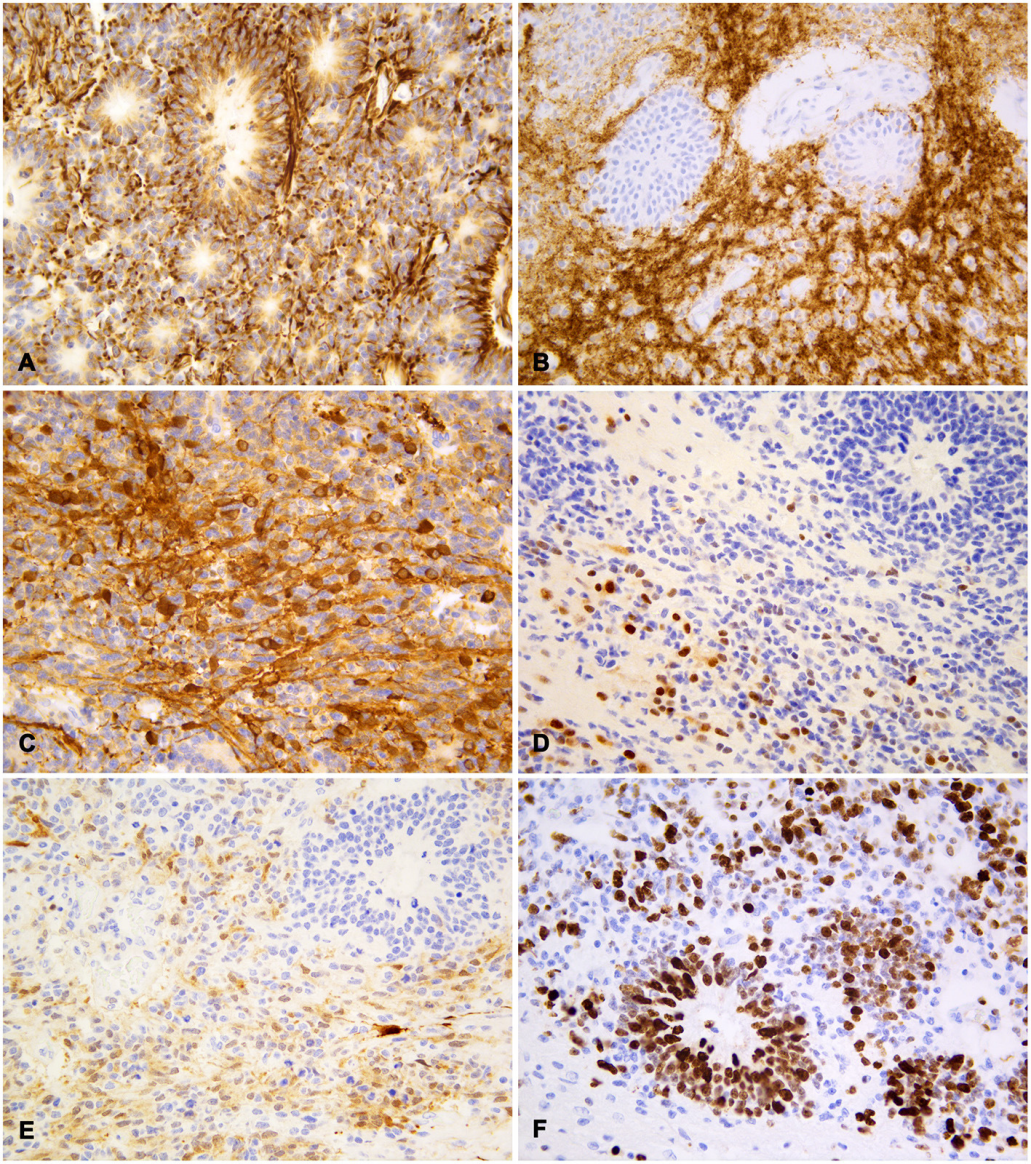
Feline embryonal tumor with multilayered rosettes, neuropil-like areas and foci of neurocytic differentiation; immunohistochemistry. **(A)** Diffuse staining with vimentin both in rosettes and in undifferentiated neoplastic cells. **(B)** Strong synaptophysin expression in neuropil-like areas with negative rosettes. **(C)** Multifocal NSE expression by undifferentiated cells and cells with long fibrillar cytoplasmic projections. **(D)** Scattered cells with strong nuclear NeuN staining, less intense in the cytoplasm of rare cells. **(E)** Patchy cytoplasmic and nuclear S-100 protein expression in neoplastic cells with variable staining intensity. **(F)** Frequent Ki67 nuclear expression in rosette-forming and undifferentiated cells. Diaminobenzidine (DAB) chromogen; Mayer hematoxylin counterstain.

Neoplastic cells were diffusely and strongly positive for vimentin, with exception of few scattered cells, mostly with neurocytic differentiation (Figure 3A). Neuropil-like areas exhibited diffuse and intense expression of synaptophysin and NSE (Figure 3B). Both markers were also multifocally expressed by clusters of small undifferentiated and neurocytic cells, with variable stain intensity, while multilayered rosettes were negative (Figure 3C). Rare, scattered nuclei of neurocytes and undifferentiated cells were positive for NeuN, generally in paucicellular areas (Figure 3D). Patchy cytoplasmic and nuclear S-100 protein expression was observed in cellular clusters, both in highly cellular and neuropil-like areas (Figure 3E). The intensity of staining was weak-to-moderate, with scattered strongly stained cells; rosettes were negative for S-100. Neoplastic cells were negative for both CK AE1/AE3 and GFAP, the latter expressed by occasional cells consistent with reactive astrocytes. Ki67 nuclear expression was intense both in multilayered rosettes and in small cell areas, with a medium proliferation index of about 70% (Figure 3F).

The localization, the histological and IHC aspects, according to the WHO CNS tumor classification of domestic animals, were consistent with an infratentorial (non-cerebellar) PNET with ependymoblastic rosettes, neuropil-like areas and foci of neurocytic differentiation.

## Discussion

In the present report, we described the morphological and immunohistochemical aspects of an infratentorial embryonal CNS tumor in an 8-month-old cat.

The main histological characteristics were the difference in cell density among different tumor areas (dense undifferentiated cells and loose neuropil-like areas), and the presence of true, multilayered ependymoblastic rosettes. The latter have been rarely described in veterinary CNS oncology (i.e., in bovine EBL) and differ from ependymal rosettes which are characterized by a single cell layer surrounding a central empty lumen (22).

The microscopic findings were consistent with the histological human variant of ETANTR, currently included in the group of ETMR (12). ETANTR is histologically defined by a distinctive biphasic aspect with hypercellular undifferentiated areas, hypocellular areas with abundant well-differentiated neuropil, multilayered rosettes and rare foci of neurocytic/ganglionic differentiation (23).

In the described case, the variability in IHC staining demonstrates the presence of tumor foci differentiating into different cell lines, as observed in human ETANTR (23). Indeed, neuroblastic tumor areas have synaptophysin and NSE-positive neuropil and NeuN-positive neurocytes. Foci of undifferentiated cells were also positive for tested neural markers as well as for the glial marker S-100. On the other hand, rosettes lacked the expression of mature neuronal and glial markers, but were strongly positive for vimentin, as reported in humans (23), which might suggest the poor differentiation of this cell population. To further investigate the possible differentiation lineage of rosette-forming cells, other precursor cell IHC markers, such as nestin, olig2, Sox10, and doublecortin, may be useful.

In addition, an ultrastructural study may have provided additional morphological insights on cell differentiation. In human ETANTR, ultrastructural analyses of tumor cells have identified microtubule-containing neuronal processes and rare neurosecretory granules (24). Cells forming true rosettes have prominent zonula adherens/intermediate junctions, whereas luminal surfaces lack differentiation (i.e., in microvilli, cilia, centrosomes), although basal bodies and abortive cilia have been identified in some cases (24, 25). In our case, luminal membranes of rosettes were frequently covered by fine eosinophilic contouring, consistent with necrotic debris and short cilia.

To our best knowledge, a single case of feline embryonal CNS tumor, diagnosed as a cerebellar medulloblastoma, has been reported. Medulloblastomas are the most common embryonal CNS tumors in domestic animals and humans, accounting for 25–30% of childhood brain tumors (2, 26). They are usually located in the cerebellar hemispheres and vermis with obstruction of the fourth ventricle (2). In our case, based on the localization of the tumor rostrally to the cerebellum, with focal infiltration of the cerebellar cortex, a diagnosis of medulloblastoma could have been hypothesized. This entity was excluded on the base of the histological aspect, since multilayered true rosettes are not a characteristic for this tumor (2, 8).

EBL, defined as a PNET with ependymal differentiation composed of multilayered rosettes and poorly differentiated small-to-medium cells, has been considered as a primary differential diagnosis (9). In the veterinary literature, EBLs have been reported in a dairy calf (27) and in a heifer (28), both localized in the fourth ventricle. EBL lacks areas of neuroblastic differentiation, do not form neuropil and are supposed to express GFAP (29). For the same aspects, an anaplastic ependymoma has also been excluded (1). Anaplastic ependymomas are densely cellular neoplasms with rare ependymal rosettes and pseudorosettes, although sporadic cases with ependymoblastic multilayered rosettes have been described in humans (22).

MEPL resembles the neuroepithelium of the embryonic neural tube and displays multilayered true rosettes (10). MEPL has been previously described in a cat with intraocular localization (30). This entity was excluded due to the absence of the characteristic pluristratified neuroepithelium arranged in papillae, tubules and trabeculae (10).

To the authors' knowledge, no cases of embryonal tumors resembling human ETANTR have been reported in domestic animals so far. A single case of retrobulbar embryonal tumor suggestive of an ETMR has been described in a 10-years-old Golden Retriever (31). However, in this case, rosettes had a Homer-Wright-type structure, without central lumen, and CK (AE1/AE3)-positive/Vimentin-negative immunophenotype suggesting a different cell differentiation (31).

Recent studies have demonstrated that the human CNS embryonal tumor variants with multilayered rosettes (EBL, central MEPL, and ETANTR) have uniform molecular signatures and comprise a single clinicopathological entity named ETMR (21). Indeed, up to 95% of these tumors share 19 miRNA cluster (C19MC) amplification on chromosome 19q13.42 (4). In our case, the term ETANTR, which characterizes the old histological PNET variant, was considered more appropriate since no specific information on molecular signatures is available for cats.

Human ETMR is an aggressive, WHO-grade IV tumor that occurs predominantly in infants under the age of 3 years (4). Nearly all tumors reside in the brain, ~70% occurring in supratentorial and 30% in infratentorial regions (12, 23) with reports of ETMRs occurring in the fourth ventricle (32). Human ETMRs mostly present as large well-demarcated tumors. On magnetic resonance imaging (MRI), they show isointense to hyperintense signal on T2, hypo- to isointense signal on T1 weighted images, frequent diffusion restrictions and cystic/hemorrhagic components (33). For the described case, due to the rapid deterioration of the patient's clinical condition, the owners declined suggested MRI.

The diagnosis of ETMR in humans relies on a combination of histopathology, FISH analysis of the 19q13.42 locus and IHC for the RNA-binding protein LIN28A IHC (12). The latter, in particular, is very useful for the identification of ETMRs, since LIN28A is rarely expressed in other brain tumor entities (4, 34). Further studies are necessary to understand whether feline embryonal tumors have similar gene expression profiles and whether LIN28A may function as a specific marker for tumors with multilayered rosettes in domestic animals.

According to the classification of embryonal CNS tumors in domestic animals, the case here described may fall into the category of non-cerebellar PNETs based on the anatomical localization (1). Indeed, the mass compressed the rostral cerebellar lobe but infiltrated it only focally. Nevertheless, non-cerebellar PNETs are defined as “histologically indistinguishable from medulloblastoma,” excluding a variety of morphologic variants (1). This classification scheme could be updated by including recently described entities in domestic animals or by expanding the PNET category to include multiple histological variants.

In the described case, the infratentorial localization of the mass within the rostral part of the fourth ventricle, with infiltration and compression of underlying brain stem and adjacent cerebellar cortex, explain the clinical presentation of recumbency and depression, rapidly worsened to stupor and coma. In addition, the obstruction of the ventricular system and the development of a moderate internal hydrocephalus may have contributed to the clinical signs.

Although extremely rare, juvenile embryonal tumors should be considered in the differentials of CNS disorders in young cats. The prevalence of embryonal tumors in cats and other domestic animals is probably understated. The implementation of MRIs and/or postmortem examinations in the routine diagnostic workflow in young animals with neurological or non-specific signs could increase the number of CNS embryonal tumor records. Collecting more cases would allow for better characterization of these rare entities by comparing morphological and molecular features with their human counterparts.

## Data availability statement

The original contributions presented in the study are included in the article/supplementary material, further inquiries can be directed to the corresponding author.

## Author contributions

GF collected the data and drafted the manuscript. MTM, AC, and MV contributed to the case interpretation and manuscript editing. EM performed the immunohistochemistry. MC performed the gross port-mortem examination. All authors contributed to the final version of the manuscript.

## Funding

Funding was provided by the Italian Ministry of Health (Research RC 2020).

## Conflict of interest

The authors declare that the research was conducted in the absence of any commercial or financial relationships that could be construed as a potential conflict of interest.

## Publisher's note

All claims expressed in this article are solely those of the authors and do not necessarily represent those of their affiliated organizations, or those of the publisher, the editors and the reviewers. Any product that may be evaluated in this article, or claim that may be made by its manufacturer, is not guaranteed or endorsed by the publisher.
